# Affecting future individuals: Why and when germline genome editing entails a greater moral obligation towards progeny

**DOI:** 10.1111/bioe.12871

**Published:** 2021-04-02

**Authors:** Davide Battisti

**Affiliations:** ^1^ Center for Clinical Ethics Department of Biotechnology and Life Sciences University of Insubria Varese Italy

**Keywords:** germline genome editing, genetic selection, parental responsibility, person‐affecting morality, procreative autonomy, reproduction

## Abstract

Assisted reproductive technologies have greatly increased our control over reproductive choices, leading some bioethicists to argue that we face unprecedented moral obligations towards progeny. Several models attempting to balance the principle of procreative autonomy with these obligations have been proposed. The least demanding is the minimal threshold model (MTM), according to which every reproductive choice is permissible, except creating children whose lives will not be worth living. Hence, as long as the future child is likely to have a life worth living, prospective parents may be allowed to use preimplantation genetic diagnosis (PGD) to select embryos with genetic diseases or disabilities. Assuming a consequentialist person‐affecting view of morality, this paper investigates whether the MTM is an appropriate tool to guide procreative decisions given the continuous development of reproductive genetic technologies. In particular, I consider germline genome editing (GGE) and I argue that its application in human reproduction, unlike PGD, should be conceived as person‐affecting towards future progeny. I claim that even if we assume the plausibility of the MTM within PGD, we are committed to accepting that a greater moral obligation towards progeny should guide procreative decisions if GGE were available. In this case, the MTM should no longer be considered an appropriate instrument to guide procreative choices. Finally, I investigate when we face this greater moral obligation, concluding that it applies only when prospective parents have already engaged in the in vitro fertilization process.

## 1 INTRODUCTION: THE COLONIZATION OF REPRODUCTION BY ETHICS

In the bioethical debate, one of the most followed lines of thought conceives the continuous development of reproductive technologies as a process of widening the range of procreative decisions of parents, rather than one of increasing the availability of preventative or therapeutic interventions towards future progeny.^1^ Thanks to in vitro fertilization (IVF), preimplantation genetic diagnosis (PGD) and prenatal testing among others, parents‐to‐be have more instruments to promote their procreative autonomy.^2^ According to this principle, competent members of society should be left to make their own decisions about how, when, and where to reproduce.^3^ Such an understanding of reproductive technologies is mostly due to the private nature of human procreation,^4^ the emotive nature of reproduction, and the perceived analogies between possible artificial interventions at the beginning of life and the eugenic theories and policies widespread in the 1870–1950 period.^5^

However, in recent years, several bioethicists have maintained that because of the greater and growing possibilities of such reproductive techniques we not only face an extension of our range of procreative choices, but we are also committed to new moral obligations towards future generations.^6^ As human reproduction is no longer something beyond our control, moral obligations towards future generations must be redefined in an unprecedented way. Paraphrasing Buchanan and colleagues, we would face a “colonization of reproduction by ethics”:^7^ according to this view, the realm of nature, namely what is beyond human control, is not static, but varies over time because of technological progress. Specifically, due to the development of new reproductive technologies, procreation no longer falls in the realm of nature, but in the field of choice and, as a consequence, of ethics. Following this line, we may say that “nature brought within human control is no longer nature.”^8^

Accordingly, some models and principles have been proposed to balance the principle of procreative autonomy with the aforementioned new moral obligations.^9^ Among them, the least demanding model proposed is the “minimal threshold model” (MTM),^10^ which grants parents very significant reproductive freedom. In this paper, I will assess whether the MTM is appropriate not only to deal with the aforementioned reproductive technologies, but also with the future possibility of applying germline genome editing (GGE) to human reproduction. In other words, I will assess how the extent of what parents‐to‐be owe their progeny will change in light of the availability of this practice.

In order to do this, I will assume a “person‐affecting” view of morality according to which an action or omission is morally wrong only if that action or omission makes things worse for, or harms, someone. Likewise, a beneficial action is morally right if that action makes things better for, or benefits, someone. Finally, any action or omission that does not make things better or worse for someone should be conceived as morally neutral and therefore permissible.^11^ This moral view can also be understood as consequentialist, since only effects count without considering other aspects of morality. However, I do not want to tie my argument to a specific notion of harm: consequently, I will refer to a very basic (and uncontroversial) conception of harm, one that encompasses the impairment of physical and mental well‐being and the curtailment of the range of opportunities reasonably accessible to an individual in order to be able to choose among a reasonable array of different life plans available to members of society. Although there may be cases in which these definitions of harm are not exactly coincident, there are nevertheless situations in which we appreciate consensus among such different perspectives. My aim in adopting these assumptions is not to argue that morality only deals with person‐affecting harms and benefits; rather, to develop an argument that may be accepted by people holding very different moral views, all of which agree that our actions must be constrained by their person‐affecting consequences.

The paper is structured as follows. In § 2, I will present the MTM arguing that such a model is the least demanding model that can be accepted from a consequentialist person‐affecting perspective. Hence, I will consider the MTM as an effective instrument guiding parental choices in the field of genetic selection with PGD. In § 3, I will present GGE and its (not yet available) application to human reproduction, and I will argue that this practice is a person‐affecting one. Then, I will claim that, if we assume the plausibility of the MTM in the field of PGD, we are committed to accepting that a *greater moral obligation* towards progeny than what the MTM proposes should guide reproductive choices in the field of GGE. In § 4, I will investigate the question of when we would face such a greater moral obligation, presenting two proposals: according to the first, GGE undermines the putative right to reproduce through sexual intercourse; the second proposal makes the weaker claim that parents‐to‐be face a greater moral obligation only when they already are in the IVF process. Supporting the latter, and after replying to a potential critique of my argument in § 5, in § 6 I conclude by claiming that, in such cases, the MTM should be considered inappropriate to guide parental decisions, in the light of the future availability of the application of GGE to human reproduction: such a practice entails, even from a consequentialist person‐affecting perspective, an unprecedented extension of our moral obligations towards progeny and, therefore, a substantial limitation to procreative autonomy.

## 2 THE MINIMAL THRESHOLD MODEL

According to the MTM, every reproductive choice is legitimate except bringing children into the world when there are good reasons to think that their quality of life will fall below an acceptable threshold. Lives below such a threshold are often termed “unworthwhile” or “lives not worth living” and are restricted to relatively rare cases where extreme suffering completely outweighs any expected positive experiences.^12^ The concept of “life not worth living” can intuitively be applied when extremely rare diseases “exhaustively determine the child's future”^13^ and render all her goals, present and future, impossible to pursue. This is most evident in cases in which a person suffers from a combination of profound cognitive and physical disabilities. In other words, reproducers have a moral obligation not to genetically select or bring to life individuals who will have lives overwhelmingly dominated by suffering that outweighs any benefits gained by living.^14^ By “selection,” I refer to the possibility of using PGD to profile genetically in vitro embryos before implantation and choosing, according to the genetic heritage, which embryo to transfer into the uterus.^15^

In light of the available information about the embryo’s or fetus’ state, we can reasonably know whether some criteria that render the life of the future person worth living are satisfied or not. Only in the latter case should we consider nonexistence preferable to existence; hence, prospective parents should be morally committed to not selecting an embryo when the future child is not going to have a life worth living.^16^ Moreover, in line with the MTM, selecting for genetic diseases or disabilities, so long as the child has a worthwhile life, should be considered permissible: according to Rebecca Bennett, a MTM advocate, “as long as we are choosing to create worthwhile lives, whether we choose a fetus who will be deaf, hearing, ‘ugly’, dyslexic, short, tall, highly intelligent, etc., is not a moral choice,”^17^ but a legitimate preference.

The MTM is in stark contrast with the principle of “procreative beneficence” (PB), primarily defended by Julian Savulescu.^18^ According to PB,

if couples or single reproducers have decided to have a child, and selection is possible, then they have a significant moral reason to select the child, among the possible children they could have, whose life can be expected, in light of the relevant available information, to have the best life or at least not worse than that of any of the others.^19^

In the near future, PGD may provide extra information not only on further genetic diseases, but also regarding polygenic traits such as intelligence and behavioral traits.^20^ Hence, such an expansion of available information should inform reproductive choices to maximize the quality of life or the well‐being of the progeny. Parents‐to‐be should not only have the moral obligation to select embryos free from genetic diseases or disabilities, but also those with expected high levels of memory, empathy or other such traits that, according to Savulescu and Kahane, could help their offspring realize whatever life plans they may come to have. For the purpose of this paper, it is worth noting that PB should be committed to claiming that it is prima facie morally wrong to select an embryo for a genetic disease or a disability D and to give birth to a child affected by D when there are other embryos, with a better‐expected quality of life, available for implantation.^21^

Nevertheless, although many people intuitively believe that using PGD to select for disability D should be considered harmful to the future child, we can easily reply by maintaining that the child affected by D is not harmed at all by the parents’ decision. Following Derek Parfit, the child affected by D was born in the only state she could have been born in, because existing with D is the only condition in which she could have existed.^22^ Therefore, through PGD, no embryos are directly affected by such a decision because the specific genetic inheritance of each embryo is the only chance they have for existence. There is no specific person harmed by the parents’ decision because the selected embryo is not made worse off by the choice to bring them to birth.^23^ The only situation in which an individual is wronged by the parents’ decision is when she is born with a life not worth living. Hence, from a consequentialist person‐affecting perspective, it seems more plausible to maintain that choosing which embryo to select within PGD, provided the expectation of generating worthwhile lives, should be conceived as a morally permissible decision, not implying any obligation for parents. PB would hence promote procreative choices that do not affect any specific individual, because it relies on the notion of impersonal harm.^24^ Since we assume a consequentialist person‐affecting morality, we cannot consider PB as morally mandatory.^25^ In other words, reproducers may decide to select the best child possible as PB requires, but they are not morally required to follow this model.

Supporting an impersonal view of harm would also be problematic in two different respects. Firstly, according to Bennett, although intuitions attract us to the notion of impersonal harm, from a person‐affecting perspective, we find it difficult to understand how something can be wrong when it does not affect the welfare of individuals: do we really care about benefit or harm that does not benefit or harm anyone?^26^ Secondly, from a more theoretical standpoint, if we accepted the notion of impersonal harm as conceived by PB—i.e. from a total‐maximizing consequentialist perspective (not an average maximizing view)—we should be morally committed to accepting what Parfit calls “the repugnant conclusion.”^27^ If what matters is to increase the cumulative totals of happiness, or whatever makes life good in any given society, then this leads to some rather unpleasant conclusions. It appears to entail a moral obligation to reproduce, since the more worthwhile lives are created, the higher this cumulative total of good things will be. Considering these rather unpleasant conclusions, we should reject PB and we have good reasons to embrace the MTM.

The MTM is certainly not uncontroversial, as it is difficult, if not impossible, to provide a clear, non‐arbitrary line dividing all cases of such worthwhile lives from all cases of lives not worth living. However, as David DeGrazia points out, it is also sufficiently clear that some lives are worth living and some are not.^28^ Setting aside religious perspectives that consider every life worth living, most of us may expect that lives with disabilities such as blindness, deafness, Down Syndrome are no doubt worthwhile, whereas lives affected by devastating diseases such as Lesch‐Nyhan Syndrome or Tay‐Sachs disease seem to be good candidates to fall within the notion of life not worth living.

In this work, I consider the MTM an appropriate instrument to guide parental choices in the field of PGD. As a consequence, PGD and the other selective assisted reproductive techniques available nowadays reduce procreative autonomy only when we are creating a life that is expected to be not worth living.

As shown in this section, this model requires very little from reproducers. However, the minimal demands required by the MTM and its philosophical justification may be accepted by a large number of people in our society, including people who embrace very different moral theories and different understandings of reproductive freedom. Certainly, according to many moral views, these minimal demands are not enough to define our moral obligations in the field of PGD and, in general, in human reproduction. My aim in this paper is not to provide conclusive reasons to embrace the MTM, but to show that, even stemming from such weak constraints—in light of the availability of GGE in human reproduction, which I will discuss in the next section—we can appreciate a significant redefinition of our moral obligations in human reproduction.

## 3 GERMLINE GENOME EDITING AS A PERSON‐AFFECTING PRACTICE

GGE is a genetic procedure that, in the near future, could effectively be applied in human reproduction in order to modify an in vitro early embryo’s DNA to avoid several genetic diseases or disabilities. Such a practice, especially through the CRISPR/Cas system, is relatively cheap and quite effective in changing monogenic traits in organisms.^29^ Furthermore, it is very precise, though not yet immune from episodes of off‐targets cuts.^30^ In other words, GGE consists of modifying embryos’ genomes before transferring one or more of them into the mothers’ womb. This technique will be more effective than PGD in cases where the odds of selecting a healthy embryo are either quite low or null. For instance, imagine that one of the partners is homozygous for a dominant genetic disorder, such as Huntington’s disease: here the risk of transmission to offspring is as high as 100%, and hence no mutation‐free embryos can be obtained through the IVF process.^31^ Moreover, imagine another couple where partners are both heterozygous for a dominant genetic disorder: the risk of transmission is as high as 75%, hence the chances of finding mutation‐free embryos are low. Another case where PGD is not effective is when both partners are homozygous for a recessive genetic disorder, meaning that they both carry two variants of the disease‐causing gene.^32^ In these cases, GGE can modify an affected embryo and allow the parents to give birth to a genetically related child free from genetic diseases. Furthermore, in the more distant future, with GGE it will be possible to avoid complex multigenic diseases in the offspring.^33^

At first glance, we can notice the similarities between GGE and PGD from the viewpoint of the reproducers: both practices can be used in IVF to transfer embryos that will have some specific traits according to parents’ preferences. However, I argue that PGD and GGE are different from a moral standpoint: while in the former we are merely *selecting* embryos for genetic characteristics that they already possess, in the latter we are *modifying* a specific embryo to change her genetic inheritance. Such a genetic modification may lead to significant effects on the future individual who will develop from the modified embryo. Here I maintain that, through the modification of an embryo’s DNA, we are dealing with the same *numerical identity* as that of the future person.^34^Generally speaking, “numerical identity” is the relation a thing has to itself and to nothing else. In order to argue that an embryo is numerically identical to the future individual developed from that embryo, I refer to what is called the “origin view,” namely the condition in which embryo A is the union of the particular pair of sexual cells from which person A_1_ will develop. Anyone who does not derive from A cannot be A_1_. Accordingly, “each person has a distinctive necessary property: that of having grown from the particular pair of cells from which this person in fact grew.”^35^ Modifying the embryo from which a future individual will develop through GGE, or refraining from doing so, is therefore a *person‐affecting* act or omission, that is, an act or an omission that could harm or benefit a specific individual.^36^ In Parfit’s words, we can also call it a “same person” choice.^37^

Through the parents’ decision to manipulate the embryo to avoid a genetic disease or disability, the future individual could be benefited or harmed compared to the situation in which the modification did not occur. Hence, an embryo’s genetic inheritance no longer appears to be something unchangeable and beyond parental control: because of the future availability of GGE, the future individual will have a number of possibilities, namely different versions of her genetic inheritance, with which she could come into existence. In this context, we are no longer dealing with a choice between either “existence” or “non‐existence” as in PGD; rather, we are choosing to change the nature of the future individual who would have been qualitatively different without such modification or with a different one. Modifying the genome of an embryo affected by a certain genetic condition to eradicate it will lead this embryo to develop into a numerically identical child who, without the genome modification, would have been affected by a genetic disease. At this point, a question arises: what is entailed by conceiving of GGE as a person‐affecting procedure?^38^ I argue that, in some cases, the availability of GGE generates new moral obligations towards progeny, even accepting the very weak constraints on procreative freedom in the field of PGD proposed by the MTM. To make my argument clear, I present two thought experiments: the case of Julia and that of Jeff‐to‐be.

*Julia’s case*

Julia is a newborn who is affected by disease X, which, though not so bad as to make her life not worth living, is likely to significantly compromise either: (a) her physical or psychological well‐being; or (b) her range of opportunities for choosing her own life plan; or (c) the possibility to develop abilities and skills necessary to pursue a reasonable range of those opportunities and alternatives; or (d) the capacities for practical reasoning and judgment that enable the individual to engage in reasoned and critical deliberation about those choices.^39^

I assume we would encounter a broad consensus among people if we consider X harmful to Julia, and, consequently, if we claim that Julia’s parents have a prima facie moral duty to cure her of X, provided that cure Y is available, effective, safe, legal, and cheap. In these circumstances, parents would have *control* over Julia’s state of health; therefore, if they refuse to cure her, Julia could reasonably complain about her parents’ decisions. If Julia were affected by an untreatable disease Z, it would be hard to claim that her parents are responsible for Julia’s state of health: in this case, Julia could not complain about her condition to her parents because a cure for Z does not exist. While the parents encounter a moral obligation to cure Julia of X, the same does not hold for Z, since moral duties strictly depend on the parents’ capabilities to cure Julia, which depend on the technological possibilities, the affordability of the treatments, etc.

*Jeff‐to‐be’s case*

Jeff‐to‐be is an in vitro embryo that is about to be implanted in his mother’s uterus in order to develop into a person called Jeff. Jeff‐to‐be is affected by a genetic mutation causing a genetic disease X_1_, which is likely to impair his physical or psychological well‐being and/or curtail the reasonable range of opportunities in the same way as X affected Julia. As in Julia’s case, Jeff’s life with X_1_ is expected to be worth living. A GGE treatment Y_1_ that solves the genetic mutation leading to X_1_ is available, effective, safe, legal, and cheap. However, the prospective parents decide to transfer Jeff‐to‐be without modifying his genome and, as a consequence, after 9 months Jeff will be born affected by X_1_.

Although this scenario may seem unrealistic for many people, it may occur for several reasons: here I present some of them starting from the most unlikely, even if philosophically interesting, and concluding with the most plausible ones. Firstly, Jett‐to‐be’s parents have sadistic personalities and they want to bring into the world a child who suffers from genetic disease X_1_.^40^ Secondly, his parents might want to have a sick child to take care of him for his whole life. Thirdly, the parents‐to‐be believe that X_1_ is not a condition that cuts off opportunities for Jeff; on the contrary, they think that X_1_ may help him to interact with a specific community to which the parents belong. Finally, the prospective parents do not know about Jeff’s genetic condition, since they did not engage in any process of genetic screening of the embryo to detect genetic diseases of Jeff‐to‐be. This last scenario seems plausible considering that, according to the ‘Assisted reproductive technology fertility clinic success rates report,’ only about 31.9% of IVF cycles reported the transfer of at least one embryo that underwent preimplantation genetic testing during 2017 in the United States.^41^

Regardless of the aforementioned possible reasons, I maintain that the prospective parents have a prima facie moral duty to treat Jeff‐to‐be because the moral reasons that in Julia’s case imply an obligation to treat her also apply in Jeff‐to‐be’s case. If we accept that in Julia’s case the parents have a moral obligation to cure, then we are also committed to accepting the existence of a moral duty to treat in Jeff‐to‐be’s case. To support this claim, I will compare the two aforementioned cases.

At first glance, we notice that Julia’s case and Jeff‐to‐be’s case are not perfectly equivalent, morally speaking, and this could raise problems for the claim just made: indeed, we encounter further elements in Julia’s case because we also need to take into account the current child’s suffering, whereas this is not the case for Jeff‐to‐be. However, all else being equal, Julia’s case seems, according to the consequentialist person‐affecting morality, to entail a moral obligation to treat for reasons that also apply in Jeff‐to‐be’s case. In both cases parental decisions are dealing with numerical identities, thus the parents’ decision not to treat them can compromise both the existing child’s (Julia’s case) and the future child’s (Jeff‐to‐be’s case) physical and psychological well‐being and/or the range of reasonable opportunities in their life. Julia and Jeff could be made worse off by the parents’ decision to avoid treating them and this is a strong reason in favor of a moral duty to treat, which is independent of Julia’s current suffering. Accordingly, in both cases a specific individual has a right to complain about their parents’ decision.^42^ Moreover, both treatments Y and Y_1_ are available, effective, cheap, legal, and safe; hence, both Julia’s and Jeff’s states of health are under the control of their parents. In light of these considerations, we can appreciate that the moral reasons that in Julia’s case imply an obligation to treat her are also found in Jeff‐to‐be’s case: both sets of parents face a similar moral obligation to treat their children.

Here an important clarification is necessary. In Julia’s case her parents are facing two options, (a) to cure her or (b) not to cure her, and only the former should be considered ethically right. Conversely, in Jeff‐to‐be’s case the parents‐to‐be are facing three options because we are not dealing with a person yet.^43^ The prospective parents’ options are: (a) to treat the embryo, (b) not to treat the embryo, (c) to decide not to implant the embryo,^44^ thus either choosing another embryo (if it is available) or giving up the pregnancy. Whereas the second option should be considered morally wrong, the third one should be considered morally permissible from a consequentialist person‐affecting perspective. Indeed, deciding not to implant Jeff‐to‐be does not affect any person because Jeff‐to‐be is not a person yet. Since no existing or future person is made worse off or better off by this choice, the third option should be considered a legitimate preference and, as a consequence, permissible from a moral point of view. Therefore, there is no moral obligation to implant Jeff‐to‐be.

Nonetheless, if reproducers decide to transfer into the uterus *that* specific embryo, then they face a moral obligation to treat the embryo first. By deciding to transfer a specific embryo, prospective parents are creating a particular individual who will have some interests not to be made worse off by the parents’ choices. In other words, prospective parents are *recognizing* that embryo as their future child. Hence, even though we are modifying an embryo that is not a person at the moment of the modification, we should treat it *as if* it were an actual person, in light of the expectation that that specific embryo will develop into a specific person. Due to the parents’ act of recognizing the embryo as their future child, the designated in vitro embryo shares not only a biological continuity but also a *moral continuity* with the future person developed from such an embryo. Again, this does not entail any obligation to implant the modified embryo or to give birth to a child. Even if they had initially consented to the modification and implantation, they could “withdraw” their consent to having that specific child at any time by deciding either to abort, or not to implant the modified embryo, or selecting and transferring another embryo. However, if prospective parents are transferring a *specific* embryo, in the same condition as Jeff‐to‐be, in order to give birth to a numerically identical individual with such an embryo, then they are morally required to treat that embryo through GGE.

To summarize, since the moral reasons that in Julia’s case imply an obligation to treat her are also found in Jeff‐to‐be’s case, we should accept a moral obligation to treat Jeff‐to‐be as well. In light of the availability of GGE techniques capable of preventing genetic diseases, prospective parents, in some cases, could be morally required to treat their future child, facing greater moral obligations towards progeny than proposed by the MTM. While in the context in which selective assisted reproductive technologies, including PGD, are the only available techniques, parents‐to‐be have no moral reasons not to select an embryo free from disease X_1_, in the context in which a GGE is available, safe, legal, and cheap, reproducers instead encounter a prima facie moral duty to treat the embryo affected by X_1_ using Y_1_. Therefore, while the MTM is an effective instrument to inform reproductive choices when only genetic selection is available, it is not the case in the context of GGE. In the next section, I assess in which specific circumstances prospective parents have this greater moral obligation.

## 4 WHEN DOES GERMLINE GENOME EDITING ENTAIL A GREATER OBLIGATION TOWARDS PROGENY?

Since, for the reasons explained above, GGE entails a greater moral obligation for prospective parents, we should explain what it means in practice to face such a moral obligation. Here it should be emphasized that my argument does not require parents‐to‐be to employ GGE and modify an embryo affected by a genetic disease, rather than simply employing PGD to select another one not affected by that disease, provided another embryo is available, or resorting to other reproductive technologies, such a gametes donation. This argument is silent about this decision, since no person is made worse off by it and therefore nobody can complain about this choice.

Rather, I argue that the availability of GGE generates moral obligations every time that reproducers are about to transfer a specific human embryo that will develop into a person suffering from genetic diseases that meet the following criteria: (a) they are such diseases that are compatible with a life worth living, but impair the child’s psychological and physical well‐being and/or significantly curtail the reasonable range of opportunities for choosing her own life plan; (b) they are diseases for which, at the moment of IVF, a safe treatment with GGE is available and legal and it is not possible to treat them effectively in vivo^45^ or after the birth of the child. Only in these circumstances can the child complain about the parents’ decision, and this generates moral reasons to limit the parents’ procreative freedom.

At this point, we should specify the cases in which this obligation arises. I advance two different proposals. The first is the “bold restriction of procreative autonomy” according to which, assuming the availability of GGE, reproducers have a prima facie moral duty to procreate through IVF and then transfer into the uterus an embryo free from those genetic diseases that meet criteria (a) and (b).

According to this proposal, every child born with such genetic diseases has the right to complain to their parents. Parents would have had the possibility to treat their child with GGE in order to avoid such genetic diseases. In reproduction through sexual intercourse, parents‐to‐be have no control over the genetic traits of the created embryo: it would have no chance of being modified without the existence of a germinal manipulation technique directly in the mother’s womb, which currently seems improbable. Therefore, parents‐to‐be would have a moral duty to reproduce through IVF, allowing the future child to be treated with GGE. The “bold restriction of procreative autonomy” proposal would hence undermine the right to procreate through sexual intercourse.

Such a proposal is quite controversial, very demanding for reproducers and hard to defend from a consequentialist person‐affecting perspective. In fact, the embryo created through sexual intercourse would never exist except as the result of sexual intercourse. Due to the specificity of human reproduction, the same embryo would have not existed in vitro, as a result of IVF, where parents could have treated it in order to avoid the genetic disease. The embryo conceived through sexual intercourse is the result of the encounter between a sperm and an oocyte at a specific time. If the parents had decided to undergo IVF, there would have been an encounter between different gametes at a different time and, accordingly, a different embryo would have been implanted. Therefore, individuals conceived through sexual intercourse, having no possibility of being genetically modified, cannot complain to the parents because they could not have avoided a genetic disease that meets criteria (a) and (b). They could not have existed except in the conditions in which they suffer from a genetic disease. The genetic inheritance that they possess is the only one they could have had, in the same way as the embryo selected through PGD. So, from a consequentialist person‐affecting perspective, also in light of the availability of the GGE to treat genetic diseases that meet the requirements (a) and (b), the right to natural procreation is not undermined. People who want to procreate through sexual intercourse do not have a moral obligation to avoid the aforementioned genetic diseases to their progeny. Therefore, the first proposal should be rejected.

A second and more reasonable proposal is the “mild restriction of procreative autonomy.” According to this, reproducers have a prima facie moral duty to transfer into the uterus an embryo without genetic diseases that meet criteria (a) and (b) only if they are already in the IVF process—that is, when in vitro embryos already exist—and if they want to have a child from one of those created embryos. In other words, given the availability of GGE, parents‐to‐be should not transfer the designated embryo, unless the embryo has first been guaranteed to be free from genetic diseases that meet criteria (a) and (b). Only in such cases is it reasonable to speak of a person‐affecting harm and benefit, because the existing embryo, which is designated to be implanted, would have the possibility of being modified to avoid genetic diseases and disabilities. Clearly, this moral obligation is not towards the embryo, which is not a person yet, but towards the future person who will develop from that specific embryo.

Thus, procreative choices should be morally limited for reproducers who are already in the IVF process, which means that they are no longer morally entitled to transfer into the uterus any embryo with an expected life worth living, as allowed by the MTM. Rather, parents‐to‐be are morally committed to transferring only those embryos that are free from genetic diseases that meet criteria (a) and (b). Specifically, prospective parents will encounter a prima facie moral obligation not to modify the designated embryo in order to have a child with a genetic disease or any condition that meets criterion (a), and for resorting to GGE if the designated embryo has a genetic disease that meets criteria (a) and (b). Therefore, parents‐to‐be should screen their embryos for diseases that meet criteria (a) and (b) and not only for such rare genetic diseases that make a life not worth living. This entails an extension of the use of genetic testing in embryos in the IVF process and, consequently, a significant extension of our moral obligations towards progeny.^46^Summarizing, whereas before the emergence of GGE, selecting an embryo with a genetic disease meeting criterion (a) used to be considered morally permissible, in light of the availability of GGE, transferring an embryo that will develop into a future person who could have been treated during the IVF process means facing the legitimate complaint of that child, hence a moral obligation to treat the embryo before transferring it into the uterus.

## 5 CHANGING THE CONTEXT, CHANGING THE OBLIGATIONS

Someone may contend that my argument is quite implausible in cases where the embryo is created in order to be treated with GGE, namely, when reproducers want to have a child with a modified genome. In these circumstances the planning process of GGE would need to start *before* performing IVF.^47^ To make this critique clearer, let us consider the case in which in a couple a partner is homozygous for the Huntington’s disease mutation and, as I wrote in § 3, thanks to GGE such a couple can conceive a genetically related child free from the Huntington gene. Suppose that they decide to have a genetically related child and for this reason they employ IVF creating one or more embryos, all of which are unfortunately affected by the Huntington mutation. As a consequence, they employ GGE to treat the genetic disease in one of the created embryos and they transfer the modified embryo into the uterus. In this case, the modified embryo is created only to be cured and it did not exist yet when prospective parents decided to use GGE to have a child. Accordingly, in the situation before IVF no moral duties to treat the future individual exist, since we cannot deal with any numerical identity. The decision to have a child through IVF and treat the embryo from which she will develop with GGE should be considered as a mere parental preference that does not encounter any moral constraint. There is no ground for any moral obligation to treat the future individual at the moment of planning GGE. The future individual’s coming into existence is dependent on the choice to use GGE in creating them in the first place where no obligations towards her exist. The child‐to‐be‐born will only be born because IVF plus GGE took place.

This critique fails to dismiss my argument. From a consequentialist person‐affecting morality, we can reasonably acknowledge that, generally, actions taken before conception cannot be considered morally good or objectionable. The reproducers’ decision to have a child directly determines the numerical identity of their future child who could not exist without that decision. Hence, no parental action, will, or desire about the future child can be subject to moral scrutiny before the embryo exists, that is, before the existence of a specific numerical identity.^48^

However, regardless of the parents’ intentions or motivation, if they are already in the IVF process, the embryo still exists and, thanks to GGE, there is the possibility to treat it, then the moral considerations change. In the post‐conception context, we appreciate the existence of an embryo that is numerically identical to the future individual. Treating the embryo with GGE will affect the future child: in this context, the parents’ decision no longer creates a brand‐new numerical identity, as in the pre‐conception context, but affects the child only in a qualitative way. As the context has changed (before the designated embryo did not exist, now it does), our moral obligations towards the future individual also arise, regardless of whether or not the designated embryo was created to be treated with GGE. It does not matter that without the parents’ decision the child would not have existed. If parents‐to‐be decide to have a child and after IVF they recognize a specific embryo as their future child, then they not only have *preferences* (which legitimately guided them prior to conception), but also *new moral obligations*. Returning to the main example, when reproducers who do not want to have a child affected by Huntington’s disease decide to employ IVF and then GGE to have a child free from the disease, they are manifesting only a preference; however, when, after the IVF process, they appreciate the existence of the designated embryo that shares a biological and, due to the will to implant it, a moral continuity with the future individual, prospective parents not only have a preference to transfer that embryo only after having treated it with GGE, but they also have a moral obligation to do so.

## 6 CONCLUSIONS

In the previous sections, I claimed that GGE, if applied to human reproduction, should be considered a person‐affecting practice, since it allows us to treat an embryo that is numerically identical to the future individual who will be born. Indeed, such a modification could make her better off or worse off than in a situation in which the modification never happened. Therefore, stemming from a consequentialist person‐affecting view of morality, I argued that, in some circumstances, the availability of GGE will generate a greater moral obligation towards progeny. However, prospective parents who decide to have a child through sexual intercourse will not face such moral obligations, but reproductive choices in this field should still be guided by the MTM. On the contrary, if reproducers are already engaged in the IVF process, and if they want to have a child from one of the created embryos, they have a prima facie moral duty to transfer into the uterus an embryo that will develop into a child free from genetic diseases that meet the following two criteria: (a) they are such diseases that are compatible with a life worth living, but impair the child’s psychological and physical well‐being and/or significantly curtail the reasonable range of opportunities for choosing her own life plan; (b) they are diseases for which, at the moment of IVF, a safe treatment with GGE is available and legal and it is not possible to cure them effectively in vivo or after the birth of the child. In other words, reproducers should not transfer an embryo, unless the embryo has first been guaranteed to be free from genetic diseases that meet criteria (a) and (b). In such cases, the MTM should be considered ethically inappropriate to deal with parental decisions even from a consequentialist person‐affecting morality.

## CONFLICT OF INTEREST

The author declares no conflict of interest.

## Footnotes

^1^
Davis, D. S. (2010). Genetic dilemmas: Reproductive technology, parental choices and children's futures. Oxford University Press.

^2^
Harris, J. (1998). Rights and reproductive choice. In J. Harris & S. Holm (Eds.), The future of human reproduction: Choice and regulation (pp. 5–37). Oxford University Press.

^3^
Robertson, J. A. (1994). Children of choice: Freedom and the new reproductive technologies. Princeton University Press.

^4^
Davis, D. S. (1997). Genetic dilemmas and the child's right to an open future. Hastings Center Report, 27(2), 7–15.9131346

^5^
Battisti, D. (2019). Genome editing: Slipping down toward eugenics? Medicina Historica, 3(3), 206–218.

^6^
Buller, T., & Bauer, S. (2011). Balancing procreative autonomy and parental responsibility. Cambridge Quarterly of Healthcare Ethics, 20(2), 268–276.10.1017/S096318011000091521435301

^7^
Buchanan, A., Brock, D. W., Daniels, N., & Wickler, D. (2009). From chance to choice. Genetics and justice. Cambridge University Press.

^8^
Ibid: 83. For a critique on this argument, see Denier, Y. (2010). From brute luck to option luck? On genetics, justice, and moral responsibility in reproduction. Journal of Medicine and Philosophy, 35(2), 101–129.10.1093/jmp/jhq00720181644

^9^
For a discussion of some of these different models and principles, see Saunders, B. (2017). First, do no harm: Generalized procreative non‐maleficence. Bioethics, 31(7), 552–558.10.1111/bioe.1236628786178

^10^
Buller & Bauer, op. cit. note 6. The MTM is similar to the version of the “Principle of procreative non‐maleficence” recently proposed in Van der Hout, S., Dondorp, W., & de Wert, G. (2019). The aims of expanded universal carrier screening: Autonomy, prevention, and responsible parenthood. Bioethics, 33(5), 568–576.10.1111/bioe.12555PMC659408830734373

^11^
By “someone” I mean only actual people, namely those that have existed, exist or who are going to exist in the actual world. See Arrhenius, G. (2000). Future generations – A challenge for moral theory. University Printers.

^12^
Harris, J. (1990). The wrong of wrongful life. *Journal of Law and Society*, *17*(1), 90–105.

^13^
Buller & Bauer, op. cit. note 6, p. 272.

^14^
Bennett, R. (2009). The fallacy of the principle of procreative beneficence. Bioethics, 23(5), 265–273.10.1111/j.1467-8519.2008.00655.x18477055

^15^
I use the term PGD because it is prevalent in the bioethical literature. However, the most recent scientific literature more commonly adopted the expression “preimplantation genetic testing” (PGT). PGT is used both to indicate PGD and “preimplantation genetic screening” (PGS). See Simpson, J. L., Kuliev, A., & Rechitsky, S. (2019). Overview of preimplantation genetic diagnosis (PGD): Historical perspective and future direction. In B. Levy (Ed.), Prenatal diagnosis (pp. 23–43). Humana Press.

^16^
The application of the MTM could also have consequences with respect to reproductive choices in the field of prenatal diagnosis: someone could argue that prospective parents who undergo prenatal diagnosis and discover that the future child will have a life overwhelmed by suffering have a moral obligation to abort her. Although this is a fascinating point, it is controversial and should be discussed elsewhere addressing concerns such as the psychological burdens of prospective parents and the moral status of implanted embryos, since undergoing prenatal diagnosis implies that the embryo has already been implanted. In this paper I want to focus solely on early embryos and assisted reproductive technologies.

^17^
Bennett, op. cit. note 14, p. 271.

^18^
Savulescu, J. (2001). Procreative beneficence: Why we should select the best children. Bioethics, 15(5–6), 413–426; Savulescu, J. (2007). In defence of procreative beneficence. Journal of Medical Ethics, 33(5), 284–288.10.1111/1467-8519.0025112058767

^19^
Savulescu, J., & Kahane, G. (2009). The moral obligation to create children with the best chance of the best life. Bioethics, 23(5) 274–290, p. 274.10.1111/j.1467-8519.2008.00687.x19076124

^20^
This statement is controversial if we consider a recent study according to which selecting embryos through PGD for polygenic traits has limited effectiveness. With this regard see Karavani, E., Zuk, O., Zeevi, D., Barzilai, N., Stefanis, N. C., Hatzimanolis, A., Smyrnis, N., Avramopoulos, D., Kruglyak, L., Atzmon, G., Lam, M., Lencz, T., & Carmi, S. (2019). Screening human embryos for polygenic traits has limited utility. *Cell*, *179*(6), 1424–1435.10.1016/j.cell.2019.10.033PMC695707431761530

^21^
D is a condition that reduces the expected quality of the future individual’s life without being bad enough to make the life not worth living.

^22^
Parfit, D. (1987). Reasons and persons. Clarendon Press.

^23^
Bennett, R. (2014). There can be no moral obligation to eradicate all disability. Cambridge Quarterly of Healthcare Ethics, 23(1), 30–40.10.1017/S096318011300041824229478

^24^
Notice that PB is not the only model that is grounded on a notion of impersonal harm. For instance, J. Harris provides a similar model in Bennett, R., & Harris, J. (2000). Are there lives not worth living? When is it morally wrong to reproduce? In D. Dickenson (Ed.), Ethical issues in maternal‐fetal medicine (pp. 321–324). Cambridge University Press.

^25^
Savulescu and Kahane argue that PB can be defended not only from an impersonal view, but also from a “wide person‐affecting” perspective (see Savulescu & Kahane, op. cit. note 19). However, Bennett replies that the latter is just another way of referring to impersonal considerations (see Bennett, R. (2014). When intuition is not enough. Why the principle of procreative beneficence must work much harder to justify its eugenic vision. Bioethics, 28(9), 447–455).10.1111/bioe.1204423841936

^26^
Bennett, op. cit. note 25, p. 452.

^27^
Parfit, op. cit. note 22, p. 381.

^28^
DeGrazia, D. (2017). Procreative responsibility in view of what parents owe their children. In L. Francis (Ed.), The Oxford handbook of reproductive ethics. Oxford University Press.

^29^
Ran, F. A., Hsu, P. D., Wright, J., Agarwala, V, Scott, D. A., & Zhang, F. (2013). Genome engineering using the CRISPR‐Cas9 system. Nature Protocol, 8(11), 2281–2308.10.1038/nprot.2013.143PMC396986024157548

^30^
Guttinger, S. (2017). Trust in science: CRISPR‐Cas9 and the ban on human germline editing. Science and Engineering Ethics, 24(4), 1077–1096.10.1007/s11948-017-9931-1PMC609705128653169

^31^
Cohen, J. (2020). Narrow path charted for editing genes of human embryos. Science, 369(6509), 1283.10.1126/science.369.6509.128332913081

^32^
Vassena, R., Björn, H., Peco, R., Guido, P., Raya, A., Sermon, K., & Veiga, A. (2016). Genome engineering through CRISPR/Cas9 technology in the human germline and pluripotent stem cells. Human Reproduction Update, 22(4) 411–419.10.1093/humupd/dmw00526932460

^33^
Savulescu, J., Pugh, J., Douglas, T., & Gyngell, C. (2015). The moral imperative to continue gene editing research on human embryos. Protein Cell, 6(7), 476–479.10.1007/s13238-015-0184-yPMC449105026113289

^34^
I assume that the embryo possesses the same numerical identity before and after genetic modification through GGE and therefore that the person who is born is numerically the same, although qualitatively different, regardless of GGE. Many people may agree that minimal changes in the embryo’s DNA, such as in the case of monogenic diseases, do not make an embryo A into a new embryo B. Consequently, no numerically different person comes into existence. However, I am aware that this claim is not free from controversy, since whether or not GGE applied to embryos causes a different person to come into being remains an open question and many other factors should be considered: for instance, the extent of the genetic change, the expression of the change in the individual’s life, when the effects of this change occur in the life of an individual, etc. On this point see: Zohar, N. (1991). Prospects for genetic therapy – Can a person benefit from being altered? *Bioethics*, *5*(4), 275–288; Elliot, R. (1993). Identity and the ethics of gene therapy. *Bioethics*, *7*(1), 27–40.

^35^
Parfit, op. cit. note 22, p. 352. Notice that this property cannot be fully distinctive, since any fertilized egg could have subsequently split and produced twins. In my discussion, I set aside the twinning cases, as they deserve further discussion.

^36^
Omerbasic, A. (2018). Genome editing, non‐identity and the notion of harm. In M. Braun, H. Schickl & P. Dabrock (Eds.), Between moral hazard and legal uncertainty (pp. 67–81). Springer.

^37^
Parfit, op. cit. note 22, p. 356.

^38^
In this work I intentionally avoid referring to GGE as a form of “therapy.” Although GGE might seem much more “therapeutic” than PGD, considering it as a therapeutic treatment is quite controversial and requires more consideration. See: Rulli, T. (2019). Reproductive CRISPR does not cure disease. Bioethics, 33(9), 1072–1082; Schaefer, G. O. (2020). Can reproductive genetic manipulation save lives? *Medicine, Health Care and Philosophy*, *23*(3), 381–386.10.1007/s11019-020-09947-232236793

^39^
For a discussion of the concept of “reasonable range of opportunities” see Buchanan et al., op. cit. note 7. According to the authors, a state of illness or disability can have a pivotal impact on the actual opportunities that an individual has to participate successfully in the dominant cooperative framework and, accordingly, to be capable of choosing among a reasonable array of different life plans available to members of society.

^40^
This bizarre and fascinating case is reported in Devolder, K., & Douglas, T. (2017). Gene editing, identity and benefit, presented at Genome editing – biomedical and ethical perspectives: An International Conference, Belgrade, August 20–21, 2017.

^41^
Centers for Disease Control and Prevention, American Society for Reproductive Medicine, Society for Assisted Reproductive Technology. (2019). 2017 Assisted reproductive technology fertility clinic success rates report. Retrieved from https://www.cdc.gov/art/reports/2017/fertility‐clinic.html

^42^
For an analysis of the notion of complaints in genetic engineering see Delaney, J. J. (2011). Possible people, complaints, and the distinction between genetic planning and genetic engineering. Journal of Medical Ethics, 37(7), 410–414.10.1136/jme.2010.03942021511971

^43^
Here I assume that Julia is already a person. Although this assumption is supported by common sense morality, it is controversial. See Giubilini, A., & Minerva, F. (2013). After‐birth abortion: Why should the baby live? Journal of Medical Ethics, 39(5), 261–263.10.1136/medethics-2011-10041122361296

^44^
Rulli, op. cit. note 38.

^45^
Here I refer to fetal therapy or fetal surgery. For a discussion of parental responsibility and fetal therapy, see Kanaris, C. (2017). Foetal surgery and using in utero therapies to reduce the degree of disability after birth. Could it be morally defensible or even morally required? Medicine, Health Care and Philosophy, 20(1), 131–146.10.1007/s11019-016-9727-027631407

^46^
Notice that my argument mainly applies when screening embryos first, and then treating them safely with GGE, will be feasible. Currently, this possibility encounters technical hurdles, since GGE should happen early, ideally, before the first cell division, that is, directly with or right after fertilization in order to avoid the risks of mosaicism. Carrying out genetic tests on embryos, such as PGD, at this stage would mean destroying the embryo. This technical hurdle makes impossible, at present, diagnosing the mutation in an embryo prior to treating it with GGE. In the future, scientific developments in genome editing techniques and preimplantation genetic testing could solve these difficulties. According to Avner Hershlag and Sara Bristow, this is desirable if GGE is to be implemented in human reproduction. On this point, see Hershlag, A., & Bristow, S. L. (2018). Editing the human genome: Where ART and science intersect. Journal of Assisted Reproduction and Genetics, 35(8), 1367–1370; Ranisch, R. (2020). Germline genome editing versus preimplantation genetic diagnosis: Is there a case in favor of germline interventions? Bioethics, 34(1), 60–69.10.1111/bioe.12635PMC697309431448423

^47^
I am indebted to an anonymous reviewer for bringing this issue to my attention. For a similar argument see Rehmann‐Sutter, C. (2018). Why human germline editing is more problematic than selecting between embryos: Ethically considering intergenerational relationships. The New Bioethics, 24(1), 9–25.10.1080/20502877.2018.144166929529985

^48^
Notice that there may be a theoretical case in which parents may have obligations when the embryo does not yet exist. Imagine that we were able to isolate during IVF the two gametes that will surely make up the embryo that is numerically identical to the future child. The decision to treat or not those gametes with GGE could be morally relevant, despite the embryo not yet existing. In this case, the numerical identity would not depend on the existence of the embryo as in the other pre‐conception cases. However, this is a controversial point and it deserves further investigation elsewhere.

